# Reactive Oxygen Species Regulation of Chemoresistance and Metastatic Capacity of Melanoma: Role of the Cancer Stem Cell Marker CD271

**DOI:** 10.3390/biomedicines11041229

**Published:** 2023-04-20

**Authors:** Francesca Beretti, Martina Gatti, Manuela Zavatti, Sara Bassoli, Giovanni Pellacani, Tullia Maraldi

**Affiliations:** 1 Department of Biomedical, Metabolic and Neural Sciences, University of Modena and Reggio Emilia, 41125 Modena, Italy; 2 Department of Dermatology, University of Modena and Reggio Emilia, 41125 Modena, Italy; 3 Department of Clinical Internal, Anesthesiological and Cardiovascular Sciences, Dermatology Clinic, Sapienza University of Rome, 00185 Rome, Italy

**Keywords:** melanoma, chemo-resistance, CD271, epithelial–mesenchymal transition, NADPH oxidase

## Abstract

BRAF mutations are present in 30–50% of cases of cutaneous melanoma, and treatment with selective BRAF and MEK inhibitors has been introduced. However, the development of resistance to these drugs often occurs. Chemo-resistant melanoma cells show increased expression of CD271, a stem cell marker that features increased migration. Concordantly, resistance to the selective inhibitor of oncogenic BRAFV600E/K, vemurafenib, is mediated by the increased expression of CD271. It has recently been shown that the BRAF pathway leads to an overexpression of the NADPH oxidase Nox4, which produces reactive oxygen species (ROS). Here, we examined in vitro how Nox-derived ROS in BRAF-mutated melanoma cells regulates their drug sensitivity and metastatic potential. We demonstrated that DPI, a Nox inhibitor, reduced the resistance of a melanoma cell line (SK-MEL-28) and a primary culture derived from a BRAFV600E-mutated biopsy to vemurafenib. DPI treatment affected the expression of CD271 and the ERK and Akt signaling pathways, leading to a drop in epithelial–mesenchymal transition (EMT), which undoubtedly promotes an invasive phenotype in melanoma. More importantly, the scratch test demonstrated the efficacy of the Nox inhibitor (DPI) in blocking migration, supporting its use to counteract drug resistance and thus cell invasion and metastasis in BRAF-mutated melanoma.

## 1. Introduction

Cutaneous melanoma remains the deadliest form of skin cancer, with a rapidly increasing global incidence [1], and 30–50% of patients carry oncogenic BRAF mutations, such as the common V600E substitution [2,3]. This mutation activates the downstream effector mitogen-activated protein kinase kinase (MEK). Therefore, selective BRAF and MEK inhibitors (BRAFi/MEKi) have been introduced [4,5,6], but patients often relapse after treatment due to the development of drug resistance.

CD271 (low-affinity nerve growth factor receptor, nerve growth factor receptor, or p75 neurotrophin receptor) is a member of the tumor necrosis factor superfamily that plays a pivotal role in determining cell life or death outcomes [7]. It has been demonstrated that the NGFR-p53 feedback loop is essential for maintaining the melanoma-initiating stem cell-like phenotype, suggesting NGFR as a potential target for developing a molecule-based therapy against melanoma [8,9]. Specific roles of the aggressive subpopulation expressing this stem cell marker have been documented in tumorigenic growth, metastatic dissemination, therapeutic resistance, and malignant recurrence [10]. Indeed, CD271-positive cells could represent the most important tumor cell population to target therapeutically, as demonstrated for other stem cell markers in different cancer cells. For instance, CD44 is overexpressed in many cancer types, including breast and colorectal cancer, and this overexpression is most prominent in the cancer stem-like cells within individual tumors being strongly associated with drug resistance and metastases [11]. Our previous studies indicated that CD271 expression within primary melanomas correlates with a more aggressive tumor phenotype and reduced patient survival [12], with other studies suggesting that CD271 expression is induced during acquired resistance to BRAF inhibition [13,14,15]. In vitro studies have demonstrated that MEKi in BRAF-mutant melanoma induces cytotoxic autophagy, followed by the emergence of CD271-expressing subpopulations [1]. Concordantly, the selective inhibitor of oncogenic BRAFV600E/K, vemurafenib, mediated resistance via increased expression of CD271 [15]. Based on these observations, CD271 could be a marker of tumor progression, and, interestingly, CD271 activation constitutes a stress tolerance mechanism within tumor cells. However, how stem cell markers, such as CD271, contribute to drug resistance still needs to be clarified.

Recently, in CD271^+^ melanoma cells, Akt3 (unlike Akt1 and Akt2) was identified as the most expressed protein sustaining cell survival [16].

Moreover, the increased presence of CD271 and its ligand NGF occurred in chemo-resistant melanoma cells, thus leading to an increase in migration [17]. The epithelial–mesenchymal transition (EMT) plays major roles during melanoma development. During melanomagenesis, EMT-transcription factors switch in their expression in a reversible manner [18]. In particular, a decrease in ZEB2/SNAIL2 levels and a rise in TWIST1/ZEB1 levels have been shown during the transition from melanocytes to malignant melanoma. Furthermore, this switch is linked to a poor prognosis for melanoma patients. The BRAF/MEK signaling pathway regulates the EMT-TF switch in normal melanocytes. Inhibition of the MAPK pathway with BRAF/MEK inhibitors reverses the switch in human melanoma cells [18].

Notably, the treatment combination of an MEK inhibitor with a BRAF inhibitor in BRAF-mutant melanoma cells caused high ROS production, both in vitro and in vivo. Drug-resistant cells showed a rise in ROS after acquiring resistance, suggesting that resistance to the BRAF inhibitor in melanoma cells is linked to oxidative stress [19].

It has recently been shown that BRAF signaling results in transcriptional upregulation of the oxidase NOX4, which promotes ROS generation [20]. We previously observed that Nox4 expression in BRAF-mutated melanoma cells is related to their metastatic progression [21]. In this study, we investigated the interplay between HGF, its receptor c-met, and an ROS source (Nox4) in early, late, and nonmetastatic melanoma patients, looking at cellular ROS modulation through the inhibition of HGF/c-met and Nox4 in vitro [21]. 

Here, we show that prolonged exposure of BRAF-mutant melanoma cells to vemurafenib treatment leads to the emergence of drug-resistant melanoma subpopulations characterized by an increase in CD271 expression with induced Akt3 and Nox4 activity and the subsequent EMT process. However, Nox inhibition resulted in increased cell death and a decrease in CD271 expression and cell migration, indicating that this treatment could be useful in counteracting drug resistance effects.

## 2. Materials and Methods

### 2.1. Cell Culture and Treatments

SK-MEL-28 cells are BRAF-mutated cells derived from primary malignant melanoma (American Type Culture Collection, Manassas, VA, USA), a kind gift from Prof. C. Magnoni, were cultured at 37 °C and 5% CO_2_ in DMEM-F12 (Sigma Aldrich, St. Louis, MO, USA) supplemented with 10% fetal bovine serum (FBS), penicillin and streptomycin, both 100 µg/mL, and 2 mM L-glutamine. 

The Ethical Review Committee of Modena Hospital (protocol number 1338/CE; 4/09) approved the use of melanoma biopsies. Biopsies were obtained from the Dermatology Unit of Modena Hospital after signed informed consent was obtained. 

Primary cells were derived from a BRAF-mutated nodular melanoma. Cells were dissociated into single cells with collagenase I immediately after surgical resection (1 mg/mL; Biochem, Nuoro, Italy) [22]. The cell suspension was filtered using a 100 µm strainer, and single cells were maintained in DMEM-F12 (Sigma Aldrich, St. Louis, MO, USA) supplemented in the same way as for SK-MEL-28.

To obtain vemurafenib-resistant cell lines, SK-MEL-28 cells were seeded in 2 ways:

- low-cell density, then maintained in complete medium containing 1 µM of vemurafenib (R1),
- normal-cell density, initially treated with 20 μM vemurafenib and then cultured in a complete medium containing 5 µM of vemurafenib (R5) for at least 4 weeks before being used for subsequent studies.

Vemurafenib-resistant primary cultures were established by adding 1 µM of vemurafenib (R1) into the complete medium of the primary culture for at least 4 weeks.

Maintenance of vemurafenib resistance was evaluated monthly via cell viability.

### 2.2. MTT Assay

Primary melanoma cells and SK-MEL-28 cells were seeded in 96-well plates, 4 replicates for each condition, at a density of 10,000 cells/well. Cells were then treated with DPI for 6 to 24 h. An MTT assay was performed as previously reported [23]. Absorbance was measured at 570 nm using a microplate spectrophotometer (Appliskan, Thermo-Fisher Scientific, Vantaa, Finland).

### 2.3. ROS Detection

To evaluate intracellular ROS levels, a dichlorodihydrofluorescein diacetate (DCFH-DA) assay was performed similarly to a previous description [23]. 

### 2.4. Cellular Extract Preparation

Cell extracts were obtained as previously described [23]. Briefly, cells were treated with lysis buffer (20 mM Tris-Cl; pH 7.0; 1% Nonidet P-40; 150 mM NaCl; 10% glycerol; 10 mM EDTA; 20 mM NaF; 5 mM sodium pyrophosphate; and 1 mM Na_3_VO_4_) with freshly added Protease Inhibitor Cocktail (Sigma Aldrich, St. Louis, MO, USA) and para-Nitrophenylphosphate (Sigma Aldrich) at 4 °C for 20 min. Lysates were sonicated, cleared by centrifugation, and immediately boiled in SDS-reducing sample buffer.

### 2.5. SDS PAGE and Western Blot

Whole-cell lysates from SK-MEL-28 cells and primary melanoma cells were processed as previously described [23]. Primary antibodies were raised against the following molecules: actin and Nox4 (Sigma-Aldrich, St. Louis, MO, USA), phospho AktS473, phospho AktT308, phospo ERK, Akt1, Akt2, Akt3, and ERK (Cell Signaling Technology, Lieden, Netherlands), Vimentin, ZEB1, and Slug (Santa Cruz Biotechnology, Dallas, TX, USA), and CD271 (BioLegend, San Diego, CA, USA).

Secondary antibodies, used at 1:3000 dilutions, were all obtained from Thermo Fisher Scientific (Waltham, MA, USA).

### 2.6. Immunofluorescence and Confocal Microscopy

For immunofluorescence analysis, cells were seeded on coated coverslips and processed. Confocal imaging was performed with a Nikon A1 confocal laser scanning microscope, as previously described [23].

Primary antibodies to detect Vimentin, CD271 (Abcam, Cambridge, UK), Nox4, and Slug (Santa Cruz Biotechnology, Dallas, TX, USA) were used following the datasheet recommended dilutions. Alexa secondary antibodies (Thermo Fisher Scientific, Waltham, MA, USA) were used at 1:200 dilutions.

ImageJ software was used to obtain three-dimensional projections. Image rendering was performed using Adobe Photoshop v.Cs6 software.

### 2.7. Migration Assay

Cell migration was measured using the wound-healing method in S and R1 melanoma cells. Briefly, 2 × 10^5^ cells were seeded in 12-well culture plates; 24 h later, sterile P-100 pipette tips were used to make artificial perpendicular scratches on the cell layer, and the floating cells were washed twice with PBS. Appropriate complete medium, with or without 0.1 µM DPI, was replaced, and the distance of migration was photographed with a microscope at different time points and quantified with ImageJ v.win64 software (US National Institutes of Health, Bethesda, MA, USA). 

### 2.8. Apoptosis Analysis

The percentage of apoptotic SK-MEL-28 cells was quantified using Caspase 3/7 cytometric flow analyses with Muse^®^ technology (Luminex Corp., Austin, TX, USA) according to the manufacturer’s instructions. Cells were trypsinized and centrifuged after treatment. The cells were resuspended in 50 μL of assay buffer BA mixed with 5 μL of Muse^®^ Caspase-3/7 reagent and then incubated for 30 min in a 37 °C incubator supplied with 5% CO_2_. Then, cells were stained with 7-aminoactinomycin D (7-AAD) for 5 min at room temperature in the dark to measure cell viability. Finally, cells were run on the Muse Cell Analyzer. 

### 2.9. Statistical Analysis

The experiments were performed three times. Values were reported as mean ± SD based on at least triplicate analysis for each sample. GraphPad Prism^®^ v.6.0 software was used to perform statistical analysis and plot layout. One-way ANOVA with the Bonferroni post hoc test or Student’s *t*-test was applied. 

## 3. Results

### 3.1. Induction of Resistance in the SK-MEL-28 Cell Line

**Figure 1:**
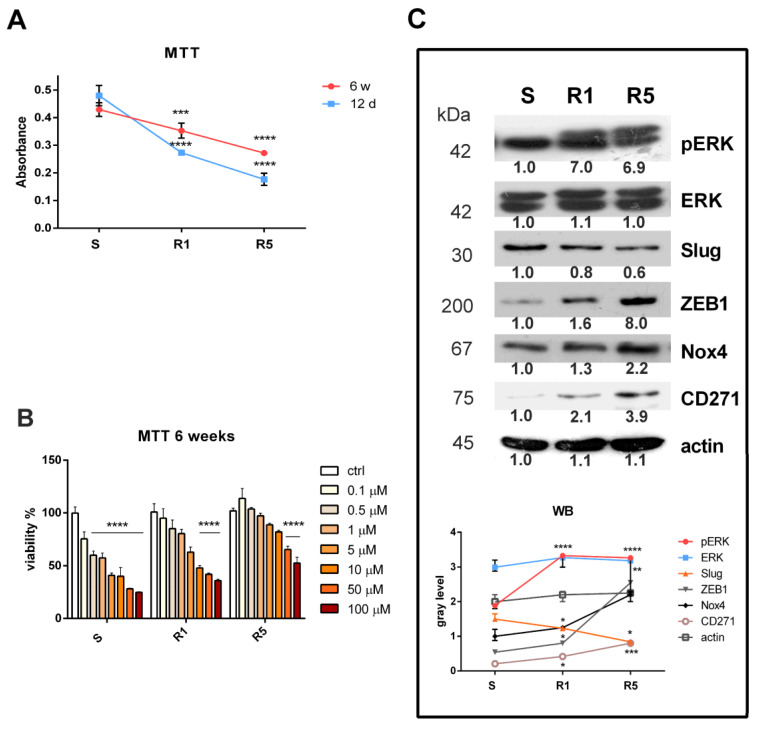
Characterization of BRAF-mutated melanoma cell line induced to resistance to vemurafenib. (**A**) Graph showing viability test with MTT assay measured in SK-MEL-28 cells induced or not induced to resistance with method 1 (R1) or method 2 (R5) for 12 days or 6 weeks. *** *p*-value < 0.001; **** *p*-value < 0.0001. (**B**) Graph showing resistance test with MTT assay measured in SK-MEL-28 cells (sensitive (S) or R1 or R5) (after 6 weeks) treated with vemurafenib from 0.1 up to 100 µM for 3 days. **** *p*-value < 0.0001. (**C**) Western blot analysis of total lysate of S, R1, and R5 SK-MEL-28 cells revealed with anti-p-ERK (R1 and R5 **** *p*-value < 0.0001), normalized to anti-ERK, anti-Slug (R5 * *p*-value < 0.05), anti-ZEB1 (R1 *p*-value < 0.05; R5 ** *p*-value < 0.01), anti-Nox4 (R1 *p*-value < 0.05; R5 *p*-value < 0.01), anti-CD-271 (R1 *p*-value < 0.05; R5 *** *p*-value < 0.001) and anti-actin, as a loading control.

Long-term treatment with vemurafenib was used to generate BRAFi resistant cells; in particular, two protocols were followed and compared with SK-MEL-28 sensitive cells as described above. R1 represents 1 µM vemurafenib-treated cells, and R5 represents a 5 µM vemurafenib-treated culture. After a long exposure time (6 weeks), resistance to cell death was visible for both R1 and R5 compared to the resistance observed after a short exposure time (12 days), suggesting that the cells developed drug resistance as they adapted to vemurafenib treatment (Figure 1A). Indeed, a drug resistance test performed on R1 and R5 after 6 weeks confirmed this data: treatment with vemurafenib from 0.1 μM up to 100 μM significantly induced cell death in sensitive cells (S), starting at 0.1 μM, while for R1, it occurred at 10 μM, and for R5 only at 50 μM (Figure 1B). Analyzing the IC50 values, we can observe that the S sample reached IC50 at 2.3 μM vemurafenib, R1 at 14.3 μM, and R5 at 94.3 μM. 

This modification was linked to changes in the expression of several proteins (Figure 1C); the activation of ERK, namely its phosphorylation, increased in R1 and R5, demonstrating that vemurafenib, which usually reduces phosphorylation of ERK, was not exerting its effect any more. The expression pattern of EMT-transcription factors, such as Slug and ZEB1, revealed an opposite trend, which is typical of melanoma cells; they could mediate a phenotype switch associated with resistance to the BRAF inhibitor. Interestingly, the expression of Nox4, as a source of ROS, and of CD271, a stem cell marker, increased similarly. Since the R5 protocol limits the number of cells available for further experiments, we decided to adopt the R1 protocol to obtain drug resistance. 

### 3.2. Nox Inhibition and Drug Resistance

**Figure 2:**
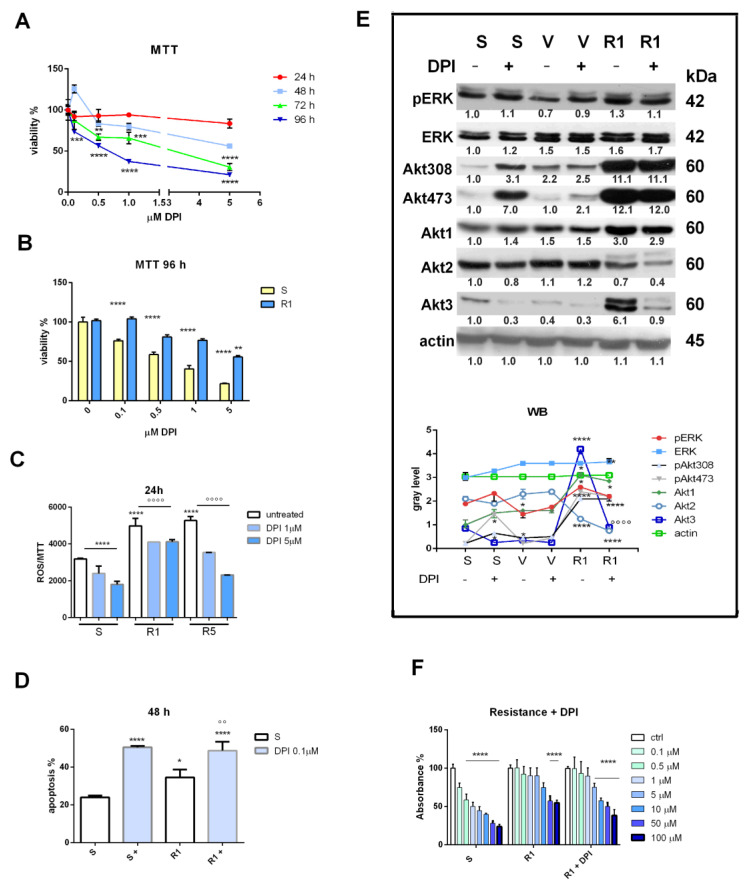
Effect of Nox inhibition on melanoma cells induced or not induced to drug resistance. (**A**) Graph showing viability test with MTT assay measured in SK-MEL-28 cells exposed to increasing concentrations of DPI for 24 to 96 h. ** *p*-value < 0.01; *** *p*-value < 0.001; **** *p*-value < 0.0001. (**B**) Graph showing viability test with MTT assay measured in SK-MEL-28 cells induced or not induced to resistance, with method 1 (R1) exposed to increasing concentrations of DPI for 96 h. ** *p*-value < 0.01; **** *p*-value < 0.0001 (**C**) Graph showing ROS levels normalized to MTT values measured in SK-MEL-28 cells S, R1, and R5 treated with DPI (+) up to 1 or 5 µM for 24 h. ****, °°°° *p*-value < 0.0001. (**D**) Graph showing apoptosis induction with MUSE assay measured in SK-MEL-28 cells (sensitive (S) or R1) treated with DPI 0.1 µM for 48 h; * *p*-value < 0.05; °° *p*-value < 0.01; **** *p*-value < 0.0001. (**E**) Western blot analysis of total lysate of S, treated with vemurafenib for 48 h (V), and R1 SK-MEL-28 cells, treated or not treated with 0.1 µM DPI for 24 h, revealed with anti-*p*-ERK (V * *p*-value < 0.05; R1 *p*-value < 0.05), anti-ERK, anti-p-Akt308 (S+ ** *p*-value < 0.01; V and V+ *p*-value < 0.05; R1 and R1+ **** *p*-value < 0.0001), anti-p-Akt473 (S+ *p*-value < 0.01; V+ *p*-value < 0.05; R1 and R1+ *p*-value < 0.0001), anti-Akt1 (R1 and R1+ *p*-value < 0.05), anti-Akt2 (R1 *p*-value < 0.05; R1+ *p*-value < 0.01; R1 vs. R1+ *p*-value < 0.01), anti-Akt3 (S+, V, V+, R1+ *p*-value < 0.05; R1 *p*-value < 0.0001; °°°° R1 vs. R1+ *p*-value < 0.0001), and anti-actin, as a loading control. (**F**) Graph showing resistance test with MTT assay measured in SK-MEL-28 cells (sensitive (S) or R1 or R1 + 0.1 µM DPI) treated with vemurafenib from 0.1 up to 100 µM. **** *p*-value < 0.0001.

In order to reduce ROS production, treatment with DPI, an inhibitor of NADPH oxidase, was evaluated. Viability tests performed on SK-MEL-28 sensitive cells showed that DPI is cytotoxic only after 96 h but already at 0.1 μM (Figure 2A). Indeed, the IC50 value after 96 h of treatment was 0.68 μM, and after 72 h, it was 1.67 μM, while for shorter treatments, the IC50 was not reached. On the other hand, R1 cells seemed to be affected by 96 h of treatment with DPI starting from 0.5 μM, but in a significant manner only at 10 μM (Figure 2B). In fact, looking at IC50 values, it was 4.95 μM, while a lower concentration (0.75 μM) was sufficient to reach IC50 for the S sample. DPI’s efficacy in reducing ROS production should be the first effect of the Nox inhibitor; therefore, an evaluation of ROS concentration was performed after a short time (24 h) using still not yet cytotoxic concentrations. Figure 2C shows that the ROS content was higher in R1 and R5 compared to S cells and that DPI was able to diminish oxidative stress in all conditions. The effect of DPI on the decrease in ROS and its cytotoxicity could be united through activation of an apoptotic pathway on both S and R1 cells, as shown in Figure 2D.

In order to dissect the mechanism underpinning the DPI-dependent ROS decrease in melanoma cells acutely or chronically exposed to vemurafenib, two classical prosurvival pathways (ERK and Akt) were investigated. Considering a short duration of vemurafenib treatment (SK-MEL-28 sensitive cells exposed to vemurafenib for 48 h), a decrease in ERK phosphorylation was evident, as expected, while R1 again displayed a rise in pERK. However, DPI was not able to significantly modulate these effects, suggesting weak involvement of the ERK pathway in the DPI effect. On the other hand, the Akt one seemed to be crucial, even if in an intricate manner. Unexpectedly, an increase in Akt phosphorylation (both in serine 473 and threonine 308) occurred in S cells treated with DPI, while in V cells, DPI did not affect the Akt pattern. Interestingly, in R1, compared to S and V, a huge increase in Akt3 and less of Akt1 occurred.

This effect was parallel to a dramatic rise in Akt phosphorylation. Notably, in this condition, DPI was able to counteract all these effects on the expression and activation of the Akt isoforms, but it was most evident for Akt3.

Overall, exposure to DPI reversed the drug resistance induced in R1, restoring the efficacy of vemurafenib to a 5 μM concentration, as shown in Figure 2F. In particular, the IC50 increase due to resistance (from 1.9 μM to 77.5 μM) was reduced again thanks to DPI exposure (IC50 32.6 μM).

### 3.3. Effect of Nox Inhibition on Cell Migration and the EMT Process

**Figure 3:**
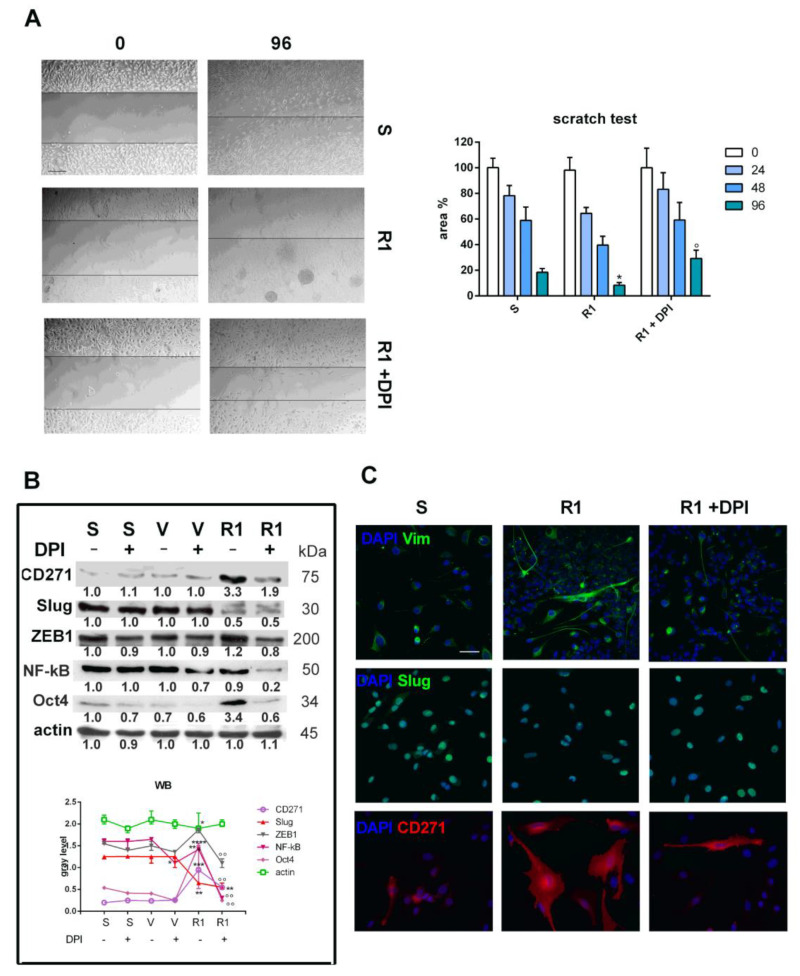
Effect of Nox inhibition on cell migration and EMT of SK-MEL-28 melanoma cells induced or not induced to drug resistance. (**A**) Representative images (scale bar = 100 µm) of migration assay obtained with scratch test measured in SK-MEL-28 cells not induced (S) or induced to resistance (R1) exposed to 0.1 µM DPI for 96 h; graph showing migration assay, namely the area not invaded by cells, after 24, 48, and 96 h (R1 * *p*-value < 0.05; R1 vs. R1+ ° *p*-value < 0.05). (**B**) Western blot analysis of total lysate of S treated with vemurafenib for 48 h (V) and R1 SK-MEL-28 cells treated or not treated with 0.1 µM DPI for 24 h, revealed with anti-CD271 (R1 *** *p*-value < 0.001; R1+ ** *p*-value < 0.01; R1 vs. R1+ °° *p*-value < 0.01), anti-Slug (R1 and R1+ *p*-value < 0.01), anti-ZEB1 (R1 *p*-value < 0.05; R1 vs. R1+ *p*-value < 0.01), anti-p50 subunit of NF-κB (V vs. V + * *p*-value < 0.05; R1 vs. R1+ *p*-value < 0.01), anti-Oct4 (R1 **** *p*-value < 0.0001; R1 vs. R1+ *p*-value < 0.01), and anti-actin, as a loading control. (**C**) Representative images with DAPI (blue), Vimentin or Slug (green), or CD271 (red) signals of SK-MEL-28 S, R1, and R1 + 0.1 µM DPI for 24 h. Scale bar = 20 µm.

To evaluate the effect of Nox inhibition on cell migration (i.e., the capacity to disseminate and cause the metastatic process), the scratch test was performed at different time points. As shown in Figure 3A, it is notable that after 96 h, R1 cells displayed greater migration activity than S cells. More importantly, DPI was able to reduce cell migration by R1 cells at all time points but in a significant manner only after 96 h.

Since EMT frequently occurs at the invasive front of epithelial tumors, destroying the well-defined epithelial structures and allowing cancer cells to migrate, invade tissues, and intravasate into blood or lymphatic vessels [24], we next investigated the regulation of two EMT-typical transcription factors (Figure 3B,C). The decrease in Slug observed in R1 compared to S cells (Figure 1C) was not modified by DPI exposure, while the ZEB1 increase was clearly reduced. The levels of these factors did not change after acute treatments (V cells). In Figure 3B, we show that Oct4 expression dramatically increased in resistant samples (R1) compared to sensitive cells (S), but DPI exposure strongly averted this effect. Interestingly, the level of the p50 subunit of NF-κB, a well-established driver of EMT in various malignancies, decreased in the presence of DPI in both R1 cells and V cells. Consistently, the induction of vimentin expression in R1, a typical marker of mesenchymal transition, diminished with DPI (Figure 3C). Notably, the huge CD271 expression in R1 cells dramatically fell in the presence of DPI (Figure 3B,C), suggesting a broad effect of Nox inhibition on different nodal points in the progression of melanoma.

### 3.4. Drug-Resistant Primary Melanoma Cells: Effect of Nox Inhibition

In order to test the therapeutic role of the Nox inhibitor (DPI) on primary cells obtained from patients, we put in culture cells derived from a BRAFV600-mutated primary nodular melanoma and repeated the most crucial experiments demonstrating DPI’s efficacy.

At first, we needed to confirm that this cell model was comparable to SK-MEL-28 S and R. Therefore, after the application of protocol 1 of drug-resistance induction, we compared these R1 cells to primary melanoma S cells and relative acute treatment (V). The panel of protein expression is shown in Figure S1. As seen with the SK-MEL-28 model characterization, CD271, Nox4, Akt3, vimentin, and pERK rise in R1, while Slug does not. Interestingly, an increase in CD271 and Akt3 also occurs, even with acute treatment (V), meaning that these are the first steps involved in Nox4 activation, resistance to vemurafenib, and then the EMT process.

**Figure 4:**
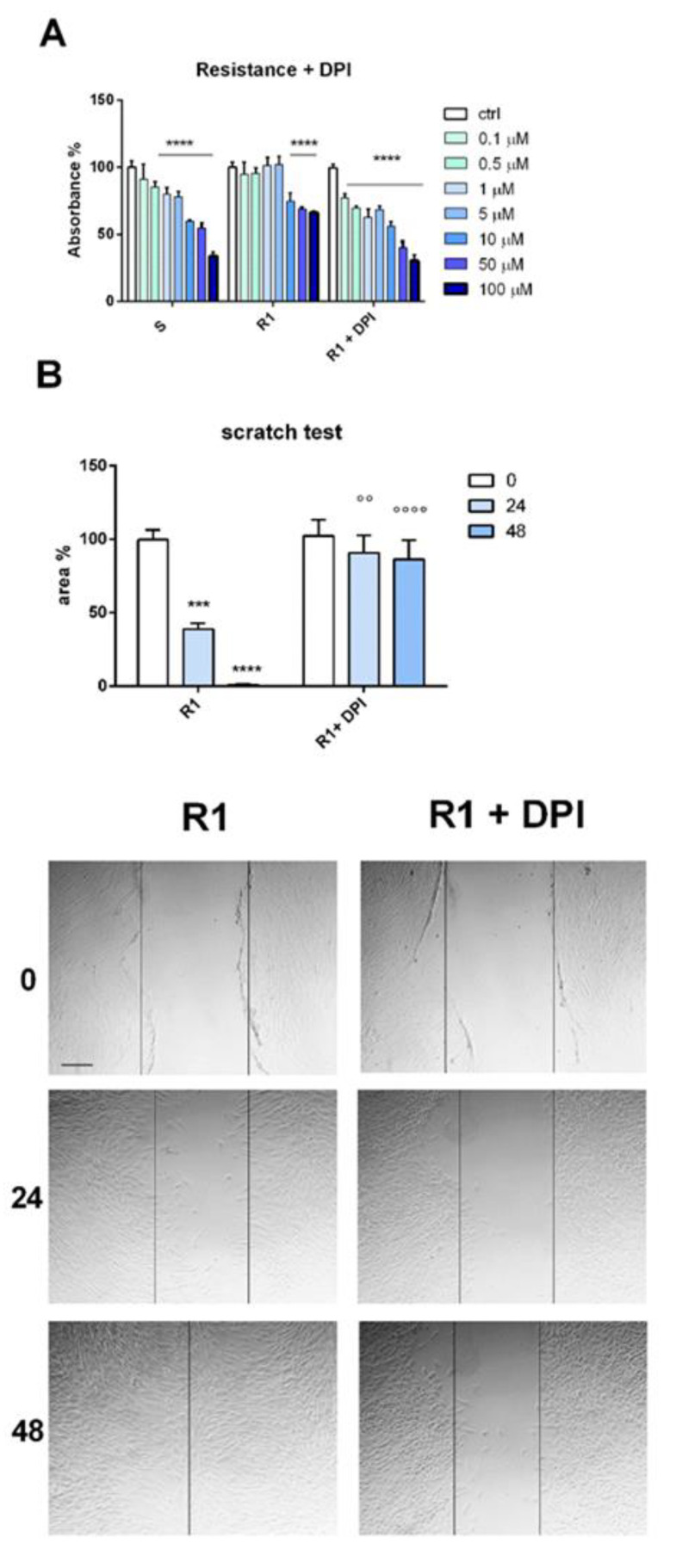
Effect of Nox inhibition on drug resistance and cell migration of primary melanoma cells. (**A**) Graph showing resistance test with MTT assay measured in primary melanoma cells (sensitive (S) or R1 or R1 + 0.1 µM DPI) treated with vemurafenib from 0.1 up to 100 µM for 3 days. **** *p*-value < 0.0001. (**B**) Graph showing migration assay, namely the area not invaded by cells, after 24 and 48 h. Representative images (scale bar = 100 µm) of migration assay obtained with the scratch test measured in primary melanoma cells induced to resistance (R1) or R1 + 0.1 µM DPI for 48 h. °° *p*-value < 0.01; *** *p*-value < 0.001; ****, °°°° *p*-value < 0.0001.

Next, resistance and migration tests were performed in primary melanoma cells (S and R1) treated or not treated with DPI. Figure 4 demonstrates again that DPI effectively increased the sensitivity of R1 cells to vemurafenib, even in a more evident manner, compared to the SK-MEL-28 case (Figure 2F). Indeed, the IC50 value was 34.6 μM for S, 139.8 μM for R1, and 14.2 μM for R1 treated with DPI. Furthermore, the capacity to stop migration was already evident starting from 24 h of DPI exposure, which supports its therapeutic potential even further.

## 4. Discussion

In this study, we established that Nox inhibition is a potential therapeutic strategy to combine with BRAF inhibitors in the treatment of melanoma patients, aiming to overcome drug resistance. Approved therapies for melanoma are often unsuccessful because resistance to BRAF and MAPK pathway inhibitors occurs. Vemurafenib is a specific mutant BRAF inhibitor; consistently, in the in vitro models used here, the SK-MEL-28 cell line and primary melanoma cells (both harboring BRAFV600) were sensitive to this drug, reaching an IC50 lower than 1 µM. After long-duration treatments with the drug (R1 and R5), cells displayed resistance, showing that the IC50 of vemurafenib was either not reached or was above 10 µM for primary cells or SK-MEL-28 cells, respectively. Mutant BRAFV600 plays a pivotal role in metastasis, since mutant melanoma cells show a higher migration capability in vitro compared to wild-type cells [17,25]. Moreover, here we showed that resistant cells migrated more efficiently than sensitive ones. Filipp et al. demonstrated that the expression of CD271 regulates melanoma cell stemness and serves as a driver of metastasis [16]. Furthermore, we previously demonstrated that, besides other markers, CD271 is suggestive of a progression and an increase in the aggressiveness of melanomas, according to their morphologies [12,26]. Verykiou et al. demonstrated that the treatment of CD271-expressing melanoma subpopulations with RNA interference and small-molecule inhibitors of CD271 slowed down the development of MEKi resistance [1]. On the other hand, it has been suggested that CD271 activation can overcome drug resistance in melanoma cells [27]. Thus, whether CD271 controls melanocyte transformation and melanoma progression remains debated. Despite SK-MEL-28 cells and primary sensitive cells weakly expressing CD271, the acquired resistance induced a huge rise in its presence, suggesting that it acts as a key regulator of the migratory phenotype, expressing transcription factors conferring metastasis and relapse, as EMT ones. High levels of ZEB1/Slug and Oct4 expression, as we showed here, certainly sustain an invasive phenotype in melanoma, including increased expression of vimentin [18,28]. In melanoma, it has been shown that ZEB1-mediated phenotype switching is associated with drug resistance (MAPK inhibitors) [29], as well as for positive Oct4 cells exhibiting stem cell-like features [30]. Moreover, upregulation of NF-κB has been found in melanomas exhibiting BRAF mutations, and it promotes EMT, suggesting a role for both MAPK and PI3K signaling in NF-κB activation [31]. Thus, EMT is increasingly understood to be involved in several cancer features, such as tumor cell stemness, tumorigenicity, resistance to therapy, and adaptation to changes in the microenvironment [24].

Suppression of the MAPK pathway by targeting BRAF fails because melanoma cells acquire alterations that stimulate the reactivation of MAPK signaling. These alterations include overexpression of receptor tyrosine kinases, including EGFR, PDGFRß, and MET [32], and maybe CD271. However, activation of the compensatory PI3K/Akt signaling cascade also occurs; indeed, Akt activation has been implicated in rendering acquired resistance to vemurafenib in BRAF-mutant melanomas [33]. Here, we noticed that, among the three Akt isoforms, Akt3 was the most influenced by the acquisition of resistance. As a result, R1 cells showed hyperphosphorylation of Akt. These data are consistent with the study by Filipp et al. (2019) [16] in which they demonstrated, in CD271^+^ melanoma-initiating cells, high upregulation of the prosurvival network controlled by Akt3.

It has been reported that ROS levels are raised after treatment with BRAF pathway inhibitors, and ROS levels continue to be high after the acquisition of resistance [19,34]. Thus, ROS may be a key downstream effector for vemurafenib-mediated Erk1/2 reactivation [35], and ROS can be involved in promoting cancer cell migration and invasion. Of the Akt isoforms, Akt3 has been shown to upregulate ROS through NADPH oxidase (Nox) activation [36]. In melanocytic lineages, Nox1, Nox4, and Nox5 are expressed; Nox4 expression is significantly higher in a subset of metastatic melanoma tumors, while in primary and metastatic melanoma tissues, Nox1 is not differentially expressed. Moreover, Nox4 and Nox1 involve different upstream signals in invasion, since Nox1 is strongly regulated by Rac1, unlike Nox4, which is downstream of the Akt pathway [35]. We previously demonstrated in BRAF-mutated patients an association between high levels of Nox4 and metastasis occurring at least 1 year after melanoma diagnosis [21]. Here, we showed that the development of resistance by both SK-MEL-28 and primary melanoma cells was coupled with an increase in Nox4 expression. Notably, the most common system to modulate Nox4 activation is an increase in its expression, unlike Nox1 and Nox2; therefore, following the expression level of Nox4 was the simplest way to demonstrate the relevance of Nox-derived ROS in this scenario without excluding a role for other Nox isoforms.

Liu-Smith et al. [35] showed that the Nox inhibitors DPI, VAS2870, and apocynin are able to kill melanoma cells in vitro, with DPI being the most potent compound and apocynin the least potent. DPI is nonspecific, so it can inhibit most of the Nox isoforms, including Nox4 and Nox1. This is convenient from our point of view, being aware that we are in front of an ROS rise due to different forms of Nox, which is why a silencing approach for Nox4 may not be sufficient to obtain similar results. However, DPI can have high toxicity [37], so we chose to use an experimental condition in which a lower concentration of DPI was able to decrease the phenotype of drug-resistant cells without causing cell toxicity. Notably, DPI has been reported to affect the ERK pathway in several kinds of cancer cells. For instance, increased proliferation and increased phosphorylation of ERK occurring in lung cancer cells exposed to growth factor activating EGFR and HER2 were inhibited by DPI incubation [38]. In bladder cancer cells, activation of the ERK/JNK-AP1 pathways was also abolished by NADPH oxidase inhibitors, such as DPI [39]. In malignant pleural mesothelioma, DPI treatment and knockdown of Nox4 attenuated phosphorylation of AKT and ERK, leading to a decrease in ROS generation and cell viability [40]. Indeed, the Akt pathway, induced in resistant cells as an alternative signal to that of MAPK, was affected by DPI exposure, not only in phosphorylation but also in the expression pattern of Ak1 and Akt3, suggesting a more profound role of Nox inhibition rather than in the modulation of protein activity. In addition to changes in the expression of Akt isoforms, the levels of transcription factors involved in EMT and, more importantly, of the receptor for NGF (CD271) were affected by DPI as well, becoming similar to those of sensitive cells. These modulations had an influence on cell behavior, again limiting the survival of R cells from vemurafenib and blocking their migration activity. Our in vitro experimental design included a DPI treatment up to 96 h (not weeks or months), so it was necessary to use it at a low concentration, but not ultralow [41]. In order to avoid DPI toxicity to other cell types, in vivo experiments for the application of combined therapy (DPI plus vemurafenib) should be performed by testing picomolar (or subpicomolar) concentrations of DPI.

## 5. Conclusions

We demonstrated that, despite its low concentration affecting neither cell viability nor inducing apoptotic pathways, DPI was able to reverse drug resistance to vemurafenib treatment, EMT switch, and migration capability via a dramatic decrease in Akt overexpression, as shown in Figure S2.

In summary, these data suggest that inhibition of Nox could block a vicious circle that involves ROS production, leading to a further increase in CD271 expression, which, in turn, reactivates MAPK signaling. Combined treatment with DPI and vemurafenib could be a good strategy for overcoming the development of drug resistance in melanoma mutated in BRAF600, limiting their propensity to migrate and invade, causing metastasis.

## Data Availability

Data sharing is not applicable to this article.

## Supplementary Materials

The following supporting information can be downloaded at: https://www.mdpi.com/article/10.3390/biomedicines11041229/s1. Figure S1: Characterization of BRAF-mutated primary melanoma cells induced to resistance to vemurafenib. Figure S2: Graphical scheme of the proposed mechanism of DPI’s effect on limiting drug resistance.
