# Supplementary material for: Reactive Oxygen Species Regulation of Chemoresistance and Metastatic Capacity of Melanoma: Role of the Cancer Stem Cell Marker CD271
Source: Biomedicines. 2023 Apr 20;11(4):1229. doi: 10.3390/biomedicines11041229 (PMC10136133; doi:10.3390/biomedicines11041229)
Supplement: Supplementary file 1 [file biomedicines-11-01229-s001.zip › biomedicines-2295673-supplementary.pdf]

Similarly to SK-MEL-28 model characterization, CD271, Nox4, Akt3, vimentin and pERK rise in R1, unlike Slug. Interestingly, the increase of CD271 and of Akt3 as well occurs even with the acute treatment (V48), meaning that these are the first steps involved in Nox4 activation and resistance to vemurafenib and then EMT process.

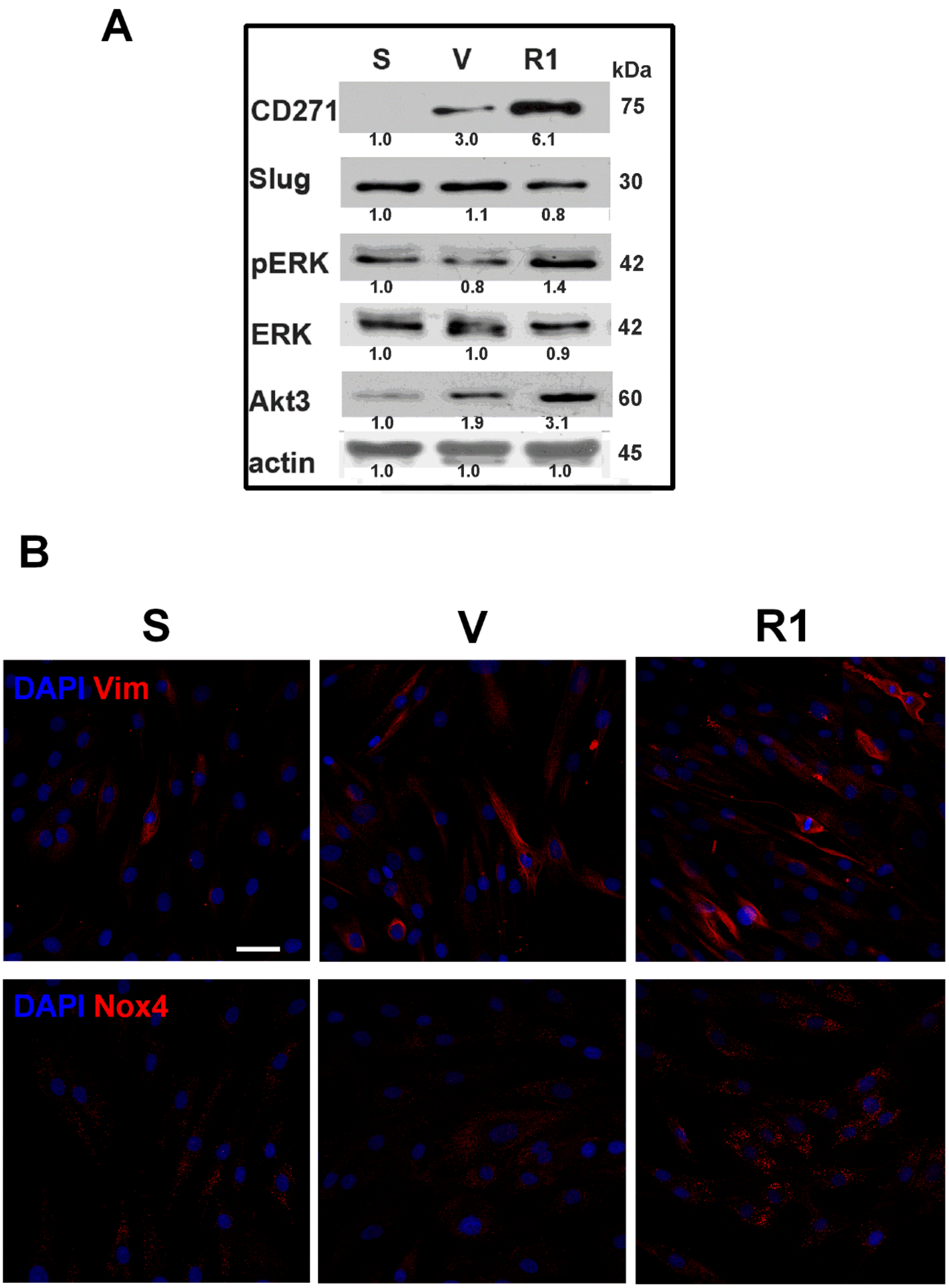

Figure S1. – Characterization of BRAF mutated melanoma primary cells induced to resistance to vemurafenib. A - Western blot analysis of total lysate of S, V and R1 melanoma primary cells revealed with anti-CD-271 (V p value < 0.05; R1 p value < 0.001), anti-Slug (R1 p value < 0.05), anti-p-ERK normalized to anti-ERK (V48 p value < 0.05; R1 p value < 0.001), anti-Akt3 (V48 p value < 0.05; R1 p value < 0.001) and anti-actin, as a loading control. B - Representative images with DAPI (blue), Vimentin or Nox4 (red) signals of melanoma primary cells S, V and R1. Scale bar = 20 μm

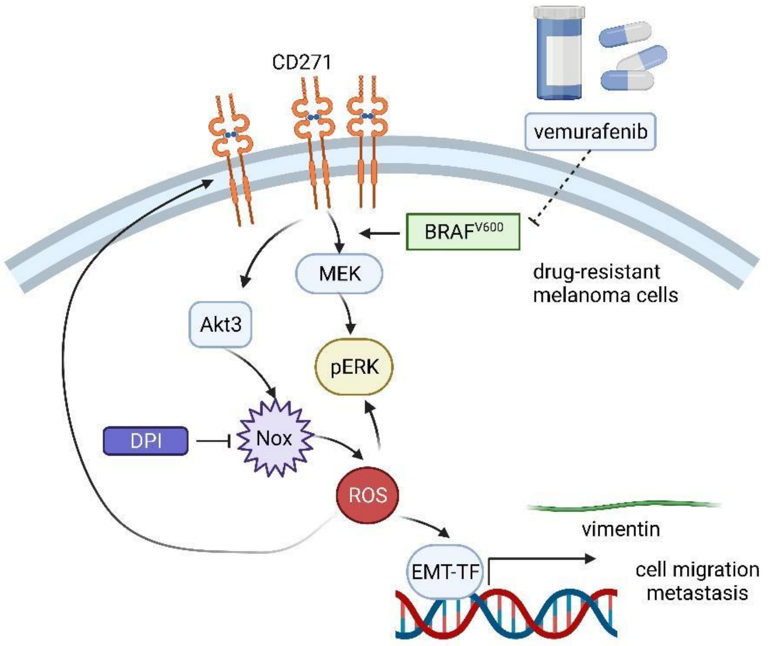

Figure S2 – Graphical scheme of the proposed mechanism of DPI effect on limiting drug resistance.

To sum up, these data suggest that the inhibition of Nox could block a vicious circle that involves ROS production leading to a further increase in CD271 expression which, in turn, re-activates MAPK signalling. The co-treatment of DPI and vemurafenib could be a good strategy to overcome the development of drug resistance in melanoma mutated in $BRAF^{600}$, limiting their propension to migrate and invade causing metastasis.
